# Caisson Disease and Vivisection

**Published:** 1904-05-21

**Authors:** Edward Berdoe

**Affiliations:** Tyneworth House, Victoria Park Gate, London, N.E.


*To the Editor of* The Hospital.

### CAISSON DISEASE AND VIVISECTION.

*To the Editor of* The Hospital.

Sir,—In your article, in your issue for May 7, on "Diver's and Caisson Disease," you inform us that Professor Leonard Hill has discovered, by experiments upon animals carried out in conjunction with Professor McLeod, the mode of causation of "diver's disease." We are informed that "he has found that the blood, when under pressure, dissolves a considerable and constantly-increasing amount of atmospheric gases, and that these tend to escape rapidly when the pressure is removed." You further say that "the whole secret of avoiding danger is to remove the compression so gradually that no effervescence is permitted to occur," and you conclude by saying "that the event adds yet one more to the long list of benefits conferred upon mankind by the physiologists for whose proceedings a bevy of old women of both sexes can find no words of adequate condemnation."

As one of these "old women" may I trespass on your courtesy so far as to be permitted to point out that in Osler's "Principles and Practice of Medicine" (Second Edition. 1895. P. 888) we read: "It has been suggested that the symptoms are due to the liberation in the spinal cord of bubbles of nitrogen which have been absorbed by the blood under the high pressure, and the condition found at the autopsies just referred to is held to favour this view." We find also the following suggestions for preventing the trouble: "In all caisson work care should be exercised that the time in passing through the lock from the high to the ordinary pressure be sufficiently prolonged." It is to be noted that the cause of the disease and its prevention were accurately explained so long ago as 1895, so that Professor Hill is hardly the wonderful discoverer in this particular instance which your article would have us "old women of both sexes" believe. For my part, I cannot understand where the animal experimentation in this connection has enlightened us, otherwise than by possibly confirming the truth of conclusions already arrived at by pathologists in the post-mortem room.

I am, Sir, yours, etc.,

Edward Berdoe, M.R.C.S., L.R.C.P.E., L.S.A.

Tyneworth House, Victoria Park Gate,
London, N.E., May 11th.

[Our correspondent does not appear to appreciate the difference between a mere supposition and an ascertained fact. The theory of causation which he quotes from Osler's well-known text-book is qualified in the same paragraph by the remark that "The explanation of this condition (diver's paralysis) is by no means satisfactory." The advance which has been made from this position of doubt by Professor Hill will, we think, be sufficiently obvious to most people.—Ed. "The Hospital."]

